# Efficacy of tetravalent coryza vaccine against the challenge of *Avibacterium paragallinarum* serovars A and B isolates from Indonesia in chickens

**DOI:** 10.14202/vetworld.2019.972-977

**Published:** 2019-07-05

**Authors:** Agnesia Endang Tri Hastuti Wahyuni, Dhasia Ramandani, Vinsa Cantya Prakasita, Sitarina Widyarini

**Affiliations:** 1 Department of Microbiology, Faculty of Veterinary Medicine, Universitas Gadjah Mada, Yogyakarta, Indonesia; 2 Department of Biotechnology and Veterinary, Vocational College, Universitas Gadjah Mada, Yogyakarta, Indonesia; 3 Department of Microbiology, Faculty of Veterinary Medicine, Universitas Gadjah Mada, Yogyakarta, Indonesia; 4 Department of Pathology, Faculty of Veterinary Medicine, Universitas Gadjah Mada, Yogyakarta, Indonesia

**Keywords:** *Avibacterium paragallinarum*, challenge test, clinical signs, serological test, vaccine

## Abstract

**Aim:**

Infectious coryza is caused by *Avibacterium paragallinarum*. In Indonesia, this infection results in a 10%-40% decrease in egg production by laying hens. This study was conducted to determine the effectiveness of tetravalent coryza vaccine contained *A. paragallinarum* bacterin serovars A, B, C2, and C3; strain A-221, B-Spross, C2-Modesto, and C-3-Akko in layers based on antibody titer and clinical signs using a post-challenge test.

**Materials and Methods:**

Forty four-week-old Lohmanns strain chickens were used in this study. Forty chickens were divided into four groups for serological and challenge test: Group 1 (unvaccinated and challenged by *A. paragallinarum* serovar A), Group 2 (unvaccinated and challenged by *A. paragallinarum* serovar B), Group 3 (vaccinated and challenged by *A. paragallinarum* serovar A), and Group 4 (vaccinated and challenged by *A. paragallinarum* serovar B). Vaccination was done using the tetravalent vaccine in oil-emulsion adjuvant contained *A. paragallinarum* bacterin serovars A, B, C2, and C3; strain A-221, B-Spross, C2-Modesto, and C-3-Akko. Vaccination was performed at day 1 and booster was done at day 14. Blood serum was collected on days 0, 14, and 28 for the hemagglutination-hemagglutination inhibition (HI) test. The challenge test was given at day 29 through intranasal administration using *A. paragallinarum* serovars A-L2447 and B-L1710 approximately 6×10^8^ CFU/mL. Clinical signs were observed for 14 days post-infection. At the end of the study, chickens were euthanized, and pathological features of the infraorbital sinus, facial skin, and trachea were recorded.

**Results:**

Data analysis of antibody titers and pathological changes was performed descriptively, while clinical symptom scores were analyzed non-parametrically with the Mann–Whitney U-test using SPSS version 21. At days 14 and 28 post-vaccination, the antibody titer in Group 3 was 5 HI and 20 HI, respectively. However, the antibody titers in Group 4 at 28 days post-vaccination were 0 HI. Clinical observations, the vaccinated groups that were challenged with *A. paragallinarum* serovars A and B showed clinical symptoms on days 4 and 6 post-infection, namely mild unilateral facial edema and severe bilateral facial edema, respectively. Clinical signs in Groups 3 and 4 were less severe than in Groups 1 and 2 (p<0.05). Pathological examination findings supported clinical observations and serological testing.

**Conclusion:**

Tetravalent coryza vaccine in chickens has efficacy to protect against the challenge test of *A. paragallinarum* serovars A and B isolated from Indonesia.

## Introduction

*Avibacterium paragallinarum* (formerly *Haemophilus paragallinarum*) is the causative agent of infectious coryza, a disease of the upper respiratory tract of chickens [1]. The clinical signs of this disease include nasal discharge, facial swelling, and lacrimation [2-5]. Infectious coryza is very important and results in poultry economic losses. The economic effect is associated with both a decrease in egg production (10%-40%) and an increase in the culling rate of laying hens [5,6]. The page classification divided *A. paragallinarum* into three serovars (A, B, and C) [4]. The Kume proposed a new serovar scheme based on the detection of hemagglutinin (HA) in *A. paragallinarum* into three serogroups (A, B, and C), which currently recognizes as nine serovars (A-1, A-2, A-3, A-4, B-1, C-1, C-2, C-3, and C-4) [4,7]. The presence of all three page serovars (A, B, and C) has been confirmed in Indonesian chickens [8]. The multiple serovars of *A. paragallinarum* and the absence of cross-protection among serovars are suspected to be the cause of frequent failures of vaccination programs [9,10]. A leading issue is that commercially marketed vaccines consist of a classic homogeneous serovar variation of local *A. paragallinarum* variants as reported in both South Africa and Thailand [11,12].

The widely used commercial coryza vaccine is a trivalent inactivated vaccine containing three strains representing the three serovars A, B, and C. The vaccines have provided satisfactory protection against the type incorporated, and vaccine failures mostly could be related to the omission of one of the serovars (e.g., serovar B) in some commercial vaccines [13]. Serovar B reported to have several important practical implications, and now compelling evidence that serovar B is a true serovar [8]. Serovars A and C do not provide cross-protection against serovar B. It is known that serovar B differs from serovars A and C [8].

The identification of potential vaccines that are challenged with *A. paragallinarum* has been proved to be the key to success in vaccination programs [12]. Poernomo *et al*. [8] showed that *A. paragallinarum* serovar B can be found in Indonesia [8] and needs to be addressed by selecting strains for vaccine production that is potentially cross-reactive, with those causing outbreaks. The new commercial coryza vaccine contains four serovars (tetravalent), including serovars A, B, C2, and C3 that were produced overseas [10,13]. In general, results of laboratory tests stated that the tetravalent vaccine is safe and effective [13].

However, the new coryza vaccine has not been tested against local isolates to verify its efficacy. Therefore, this study was conducted to determine the effectiveness of tetravalent coryza vaccine contained *A. paragallinarum* bacterin serovars A, B, C2, and C3; strain A-221, B-Spross, C2-Modesto, and C-3-Akko in layers based on antibody titer and clinical signs using a post-challenge test.

## Materials and Methods

### Ethical approval

Research using animals was approved by the Ethical Committee from Integrated Research and Testing Laboratories, Universitas Gadjah Mada, Indonesia with the number 00043/04/LPPT/IV/2017.

### Experimental design

This research consisted of several stages: Chicken maintenance, vaccine application, blood sample collection, serology tests of *A. paragallinarum* isolates, challenge test, observation of clinical symptoms, necropsy, and observation of pathological changes. A total of 40 female 4-week-old laying hens used for this study were randomly divided into four groups of ten hens in each group. The treatment group consisted of: Group 1 (unvaccinated and challenged by *A. paragallinarum* serovar A), Group 2 (unvaccinated and challenged by *A. paragallinarum* serovar B), Group 3 (vaccinated and challenged by *A. paragallinarum* serovar A), and Group 4 (vaccinated and challenged by *A. paragallinarum* serovar B).

### Vaccination

Vaccination was done using commercially inactivated tetravalent vaccine IC Quadro (Phibro^™^) containing *A. paragallinarum* bacterin serovars A, B, C2, and C3; strain A-221, B-Spross, C2-Modesto, and C-3-Akko in oil-emulsion adjuvant. The vaccine contained at least 10^8^ inactivated cells of each strain per dose. Vaccination was done by intramuscular injection on day 1 and booster at day 14 (Groups 3 and 4). Placebo injection was performed using 0.3 ml buffered NaCl to unvaccinated chickens (Groups 1 and 2).

### Serological characterization

Blood sampling was collected on day 0 (before vaccination), day 14 (after the first vaccination), and day 28 (after booster vaccination), based on antibody response against *A. paragallinarum* vaccination in chicken as previously mentioned by Wambura [14]. Subsequently, serum was used for serological testing using the hemagglutination-hemagglutination inhibition (HA-HI) method.

#### HI test

The HI test was conducted using four HA units of antigen and 1% glutaraldehyde-fixed chicken red blood cells (GA-RBCs), as previously described [15-17]. Forty-microliter volumes of doubling dilutions of antisera at 1:5 to 1:1280 were prepared in phosphate buffer saline-bovine serum albumin-gelatin. An equal volume of antigen contained four HA units, followed by 40 µl of 1% GA-fixed RBCs, was added to each well. Plates were read after 1 h at room temperature. The titers were expressed as the reciprocal of the highest dilution of a serum sample that showed complete inhibition of the hemagglutinating activity [15-17].

### Challenge methods

The challenge test was done by intra-sinus inoculation using 0.3 ml in broth culture of *A. paragallinarum* isolate serovars A-L2447 and B-L.1710 at day 29. The challenge dose contained approximately 6×10^8^ cfu/mL. Observation of clinical symptoms and mortality of chickens was performed after the challenge and was both conducted and recorded 2 times per day for 14 days. Clinical symptoms were recorded daily and included sneezing, nasal exudate, facial swelling, snoring, and conjunctivitis, as previously described [13,18,19].

Zhao *et al*. [11] scored the results of observations based on both the presence and severity of clinical symptoms as follows: Score 0 (negative), no clinical signs; Score 1 (mild clinical signs), characterized by nasal discharge with or without mild facial edema; Score 2 (moderate clinical signs), characterized by nasal discharge on both sides accompanied by moderate facial edema; and Score 3 (severe clinical signs), characterized by severe bilateral edema with or without hemorrhage and conjunctivitis.

### Statistical analysis

The antibody titers and pathological changes were done descriptively, while clinical symptom scores were analyzed non-parametrically with the Mann–Whitney U-test using SPSS, Version 21.0. (Armonk, NY: IBM Corp.).

## Results and Discussion

The efficacy of the vaccine is known based on both serology and the challenge test. The serological test was performed to evaluate the vaccination program. Table-1 indicates that the HI test on day 0 shows that the antibody titer of all chickens is 0. On days 14 and 28, the antibody titer of Group 3 was higher than that of Group 4. Thus, on both days 14 and 28, Group 3 had higher antibody titers than those of Group 4. Based on the HI test results, Group 3 showed that the tetravalent vaccine was able to produce good antibody titers. The second vaccination (at day 14) in Group 3 showed a protective HI titer. Garcia *et al*. [20] demonstrated that over 90% of vaccinated chickens have titers more than 5 HI if the chickens receive more than one vaccination.

**Table 1:** Results of the hemagglutination inhibition test post-vaccination.

Days	HI result	Group			

		1	2	3	4
0	Pre-vaccination	0	0	0	0
1	First vaccination				
14	Days 14	0	0	5	0
14	Booster				
28	Days 28	0	0	20	0

1(Unvaccinated and test using HA antigen serovar A), 2(unvaccinated and test using HA antigen serovar B), 3(tetravalent vaccine and test using HA antigen serovar A), and 4(tetravalent vaccine and test using HA antigen serovar B). HA=Hemagglutinin

In Group 4, no antibodies were detected on days 14 and 28. However, it is not known whether these negative results in Group 4 indicate that vaccination confers no protection. Serological test results cannot be used as the only data to determine possible vaccines. The potential clinical utility of the tetravalent vaccine can also be established from the results of the challenge test [21].

HA protein, an important virulence factor of *A. paragallinarum*, plays a significant role in the adherence to host cells since adherence is the first step of *A. paragallinarum* infection. *A. paragallinarum* has a small amount of HA and thus proved to be less virulent [22]. *A. paragallinarum* serovar B has low virulence [5] and may lead to low HI titers in the inhibition HA test whenever it is performed [5].

The inactive coryza vaccine will produce an immunoglobulin titer or antibody by complex mechanisms [9,18,22]. The inactive coryza vaccine will be recognized by both B cell receptors and T cells, whose mechanism involves various cells (APC, MHC, CD4, and CD8). Chickens that are immunized with the inactive coryza vaccine have been able to generate a specific immune response 2 weeks after initial exposure. It is reported that the administered coryza vaccine provides protection up to 56 weeks after vaccination with the highest level of protection being afforded if a double dose of the vaccine is used [10,13,18,19].

Research conducted by Garcia *et al*. [20] mentions that the challenge test is the gold standard to determine the immunological response in chickens that have been vaccinated with a double dose of *A. paragallinarum*. Based on the challenge test, both the severity of symptoms and pathological changes will be known. The challenge test in this study was done through intranasal administration of *A. paragallinarum* isolates to enhance its pathogenicity.

**Figure-1:**
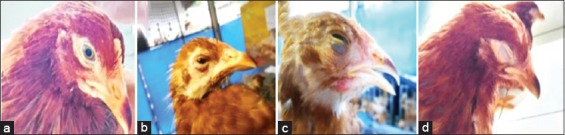
The observations of post-challenge clinical signs. (a) Control (no clinical signs); (b) mild nasal discharge; (c) moderate bilateral facial edema; (d) severe facial edema, conjunctivitis, and hemorrhage.

Infectious coryza is a disease in poultry that warrants suspicion as indicated by the results of observations of post-challenge clinical symptoms in this study (Figure-1). The development of clinical symptoms such as nasal discharge, facial edema, and conjunctivitis can be observed on the 7^th^ day after infection. Artificial infection with *A. paragallinarum* is capable of causing high morbidity that can potentially cause high losses in the cultivation of laying hens. Based on the results of observations of clinical symptoms after the challenge test, it was found that there was a difference in the number of infected chickens between both vaccinated groups (Group 3 and Group 4) and unvaccinated groups (Group 1 and Group 2). Furthermore, a group of infected unvaccinated chickens, Group 1, had the fewest number of chickens with no clinical symptoms probably because serovar A is more pathogenic than serovar B [5,22].

**Figure-2:**
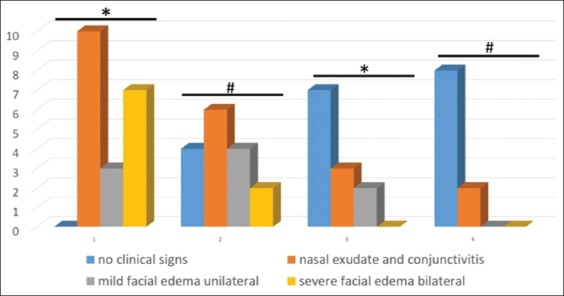
Graphic representation of the clinical signs obtained from vaccinated and unvaccinated chickens challenged with *Avibacterium paragallinarum* serovars A and B 14 days post-infection. Mild facial edema unilateral was found in Group 2, Group 3, and Group 4 compared to Group 1 (p<0.05). Group 2 showed severe bilateral facial edema compared to Group 1 (p<0.05) */#p<0.05.

Based on clinical observations, the vaccinated groups that were challenged with *A. paragallinarum* serovars A and B showed clinical symptoms on days 4 and 6 post-infection. On the other hand, the unvaccinated group began to show clinical symptoms on day 3 post-infection (Figure-2). Thus, in this study, the incubation period of *A. paragallinarum* is 3 days post-infection. Facial areas that experienced swelling were filled with thick white exudate and foul-smelling discharge. Facial swelling can be both unilateral and bilateral, depending on the severity of the disease [1,18,19,23]. Mild facial edema unilateral was found in Group 1, Group 2, Group 3, and Group 4 with a percentage of 80%, 40%, 20%, and 0%, respectively (p<0.05). Group 1 and Group 2 showed severe bilateral facial edema with a percentage of 70% and 20%, respectively (p<0.05). The difference in the severity of observed clinical symptoms proves that the potential of the given tetravalent vaccine can reduce the severity of the disease. In groups of vaccinated chickens, the number of chickens showing fewer symptoms was less than that of unvaccinated chickens.

**Figure-3:**
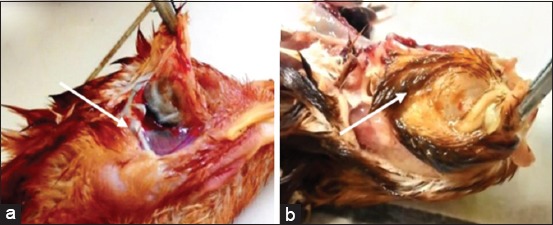
Pathological features of the facial dermis 14 days after infection: (a) Facial dermis of vaccinated chicken groups (no clinical signs found); (b) facial dermis of unvaccinated chicken groups (edema and mucopurulent exudate can be found subcutaneously).

**Figure-4:**
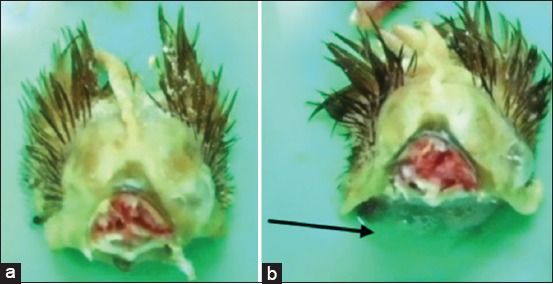
Pathological feature of infraorbital sinus 14^th^ days after infection: (a) Infraorbital sinus of the vaccinated chicken group did not found macroscopic lessons; (b) infraorbital sinus of the unvaccinated chicken group shown mucopurulent discharge.

Pathological examination was done on day 14 post-infection. Macroscopic features included watery eyes and hyperemia, and nasal discharge in the infraorbital sinus (Figure-3), edema of facial skin (Figure-4), and hyperemia of tracheal mucous membranes. Groups of vaccinated chickens were challenged with *A. paragallinarum* serovars A and B (Group 3 and Group 4), and no chickens showed signs indicating macroscopic changes. Groups of unvaccinated chickens were challenged with *A. paragallinarum* serovars A and B (Group 1 and Group 2) and some chickens showed macroscopic changes with varying severity.

**Figure-5:**
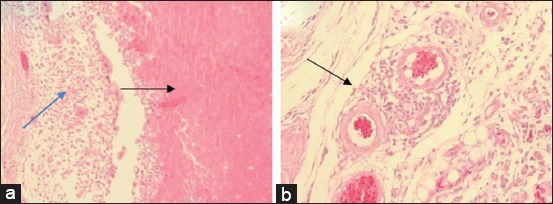
Facial dermal histopathological features from a group of unvaccinated chickens: (a) Infiltration of inflammatory cells and dominant heterophils (blue arrow), and caseous necrosis of the dermis layer (black arrow); (b) infiltration of inflammatory cells and dominant heterophils in the perivascular area (black arrow) (H and E, 400×).

**Figure-6:**
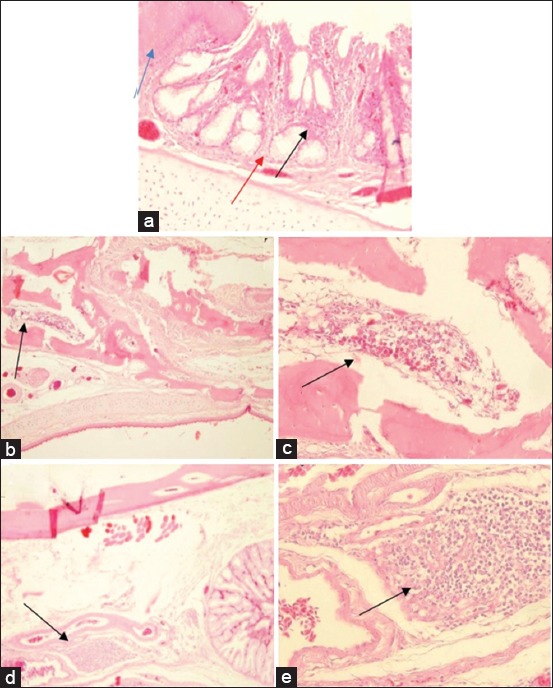
Histopathological features of an infraorbital sinus from unvaccinated chickens: (a) Proliferation of mucosal epithelium (blue arrow), hypertrophy of mucosal gland (red arrow), and infiltration of inflammatory cells and dominant heterophils in the submucosa (black arrow) (H and E, 200×); (b) infiltration of inflammatory cells and dominant heterophils in the lumen of the sinus (H and E, 200×); (c) infiltration of inflammatory cells and dominant heterophils in the lumen of the sinus (H and E, 400×); (d) perivasculitis in the submucosa of the salivary gland (H and E, 100×); (e) infiltration of inflammatory cells and dominant heterophils in the perivascular area (H and E, 400×).

Chickens in unvaccinated groups that were challenged with serovar A (Group 3) had the most severe histopathological changes in their facial dermis that was characterized by caseous necrosis of dermis layer, infiltration of inflammatory cells, and dominant heterophils in the perivascular area (Figure-5). Histopathological changes were observed in the infraorbital sinus and were characterized by mucosal epithelial proliferation, mucosal glandular hypertrophy, submucosal necrosis, and heterophilic infiltration in the lumen of the infraorbital sinus (Figure-6), as previously described [5,24,25]. These authors also report that chickens infected with coryza have clinical symptoms such as catarrhal to fibrinopurulent exudate in the nasal cavity, infraorbital sinus, and conjunctiva, and subcutaneous edema of the facial and wattle areas. The upper trachea may also be infected, but the parabronchial lungs and air sacs develop clinical symptoms in cases of chronic complications [5]. Microscopic examination in this study supports both macroscopic observations and clinical symptoms.

## Conclusion

This study demonstrated that the antibody titer from vaccination pre-challenge with tetravalent coryza vaccine protects against HA antigen serovar A but does not protect against HA antigen serovar B. Clinical symptoms and pathological changes in the vaccinated group challenged with *A. paragallinarum* serovars A and B were less severe than those in the unvaccinated group.

## Authors’ Contributions

AETHW and SW contributed to the conception and design of the study. Analysis and interpretation of the data, drafting of the article, and statistical expertise, conducted by SW and DR. Samples collection and assembly of data by DR and VCP. Finally, critical revision of the article for important intellectual contents and final approval of the article done by AETHW, SW, and DR. All authors read and approved the final manuscript.
